# Cellular Components of the Blood-Brain Barrier and Their Involvement in Aging-Associated Cognitive Impairment

**DOI:** 10.14336/AD.202.0424

**Published:** 2025-07-01

**Authors:** Kaiyuan Shen, Yi Shi, Xin Wang, Susan WS Leung

**Affiliations:** ^1^Department of Neurology, Zhongshan Hospital, Fudan University, Shanghai, China.; ^2^Institute of Clinical Science, Zhongshan Hospital, Fudan University, Shanghai, China.; ^3^Key Laboratory of Organ Transplantation, Zhongshan Hospital, Fudan University, Shanghai, China.; ^4^Department of Pharmacology and Pharmacy, Li Ka Shing Faculty of Medicine, The University of Hong Kong, Hong Kong SAR, China.

**Keywords:** components, blood-brain barrier, aging, cognitive impairment

## Abstract

Human life expectancy has been significantly extended, which poses major challenges to our healthcare and social systems. Aging-associated cognitive impairment is attributed to endothelial dysfunction in the cardiovascular system and neurological dysfunction in the central nervous system. The central nervous system is considered an immune-privileged tissue due to the exquisite protection provided by the blood-brain barrier. The present review provides an overview of the structure and function of blood-brain barrier, extending the cell components of blood-brain barrier from endothelial cells and pericytes to astrocytes, perivascular macrophages and oligodendrocyte progenitor cells. In particular, the pathological changes in the blood-brain barrier in aging, with special focus on the underlying mechanisms and molecular changes, are presented. Furthermore, the potential preventive/therapeutic strategies against aging-associated blood-brain barrier disruption are discussed.

## 1. Introduction

In 2020, the World Health Organization reported that by 2050, the proportion of the world's population over 60 years would reach 2.1 billion, which nearly doubles from 12% to 22% (https://www.who.int/news-room/fact-sheets/detail/ageing-and-health). Aging is an independent risk factor for cardiovascular and neurological diseases; as such, it poses societal challenges. With aging, endothelial and neurological dysfunction progress, significantly contributing to pathological conditions [1-3].

The central nervous system is considered an immune-privileged tissue due to the exquisite protection provided by the blood-brain barrier (BBB). The BBB is the primary site to protect the central nervous system from pathological stimuli [4]. Vascular endothelial cells, pericytes, as well as the end-feet of astrocytes are major components of the BBB. The study using recently developed single-cell sequencing and flow cytometry techniques reports that the brains of aged mice, compared to those of young counterparts, have a higher prevalence of endothelial cells and microglia, but not neurons, with high senescence gene enrichment scores [5]. In addition, lymphocytes are detected and significantly increased in the brains of 12-month-old mice when compared to younger ones [6], implying that the breakdown of the BBB occurs during aging [7]. Using an advanced dynamic contrast-enhanced magnetic resonance imaging approach in the living human brains, early vascular leakage during normal aging is observed in the CA1 and dentate gyrus regions of the hippocampus, but not in other parts of the brain regions [8], and is accompanied by an increased level of soluble platelet-derived growth factor receptor β (PDGFRβ), a cell surface marker of pericyte, in the cerebrospinal fluid [7, 8]. It indicates the loss of BBB integrity in the process of aging in both animal and human studies, and aging-associated cerebromicrovascular dysfunction plays a causal role in the development of cognitive impairment.

Therefore, the present review focuses on the pathological changes in the BBB in aging, including those in endothelial cells and pericytes, as well as in astrocytes, perivascular macrophages, and oligodendrocyte progenitor cells of the BBB. Specifically, the molecular changes in each cell types accounting for the pathological outcome are reviewed. Furthermore, potential therapeutic strategies for preserving BBB integrity during aging are discussed.

## 2. Cell components of the BBB and their interaction in the maintenance of the integrity of BBB

It is well-established that the blood-brain barrier is composed of endothelial cells, pericytes and astrocytes. For a long time, the central nervous system is considered to be devoid of immune cells other than microglia. With the technological advances, a group of macrophage-linage cells surrounding arterioles in the BBB has been identified [9, 10], and they are termed perivascular macrophages. The cells forming the BBB interact not only with each other, but also with the neighboring neuronal cells and oligodendrocyte progenitor cells (OPC), to regulate the function of the BBB. As such, dysfunction of any one type of these cells would have an impact on the integrity of the BBB.

### 2.1 Vascular endothelial cells

Endothelial cells lie in the innermost layer of the vascular wall and play a crucial role in regulating vascular tone, preventing platelet aggregation and leukocyte adhesion, and regulating cell proliferation [11-16]. The BBB endothelial cells are vital for the anatomical structure. They have very tight junctions and are tightly adhered to the extracellular matrix components of the basement membrane. Integrins are transmembrane receptors in the endothelial cells binding to the extracellular matrix [17]. Under hypoxia and pathological conditions, brain endothelial integrin α5β1 and its ligand fibronectin, a major component of basement membranes in the BBB, are upregulated [18], and their interaction is important for angiogenesis [19]. An increased fibronectin/α5β1-interaction, which occurs following exposure to the inflammatory cytokine interleukin (IL)-1, modifies the localization of the tight junction protein claudin-5 in human brain microvascular endothelial cells, and promotes the transendothelial migration of peripheral blood mononuclear cells in an *in vitro* BBB model [20]. Deletion of endothelial α5β1 integrin in mice compromises vascular integrity and impairs vascular remodeling following immunization [21], although administration of a pharmacological inhibitor of α5β1 integrin appears to be neuroprotective against ischemic stroke injury induced by transient tandem ipsilateral common carotid artery/middle cerebral artery occlusion in mice [22]. By specifically deleting the autophagy-related protein Atg-7 in endothelial cells in mice, the expression of fibronectin is downregulated, leading to BBB leakage and reduced adhesion of astrocytes to brain microvessels [23].

In endothelial cells, glycolysis is the principal energy supply [24]. The glucose transporter Glut1 and hexokinase 2, the first rate-limiting enzyme of glycolysis, are located in the abluminal side of the BBB, creating a concentration gradient of glucose to facilitate the influx of glucose from the blood into the brain [25]. During the conditions requiring increased glycolysis, the Glut1 protein translocates to the luminal side, leading to increased glucose uptake [26]. Single-cell transcriptome analysis demonstrates that the expression of Glut1 in endothelial cells of aged mouse brains is reduced compared to that in young adult mouse brains; in particular, the downregulation of Glut1 expression in the brain is also observed in patients with Alzheimer's disease [27].

It is well-known that nitric oxide is a major endothelium-derived relaxing factor, which counteracts the effects of vasoconstrictors and inhibits the production of endothelium-derived contracting factors, a major one being endothelin-1 [12-16]. Nitric oxide is also inactivated by superoxide anions. Thus, reduced nitric oxide production and elevated levels of oxygen-derived free radicals are team players in vascular disorders [28, 29]. Consistent with macrovascular endothelial dysfunction in aging, reduced vasodilatation has been reported in aged mice [30, 31], as well as in human and mice with cognitive impairment [32]. Endothelial dysfunction is associated with reduced nitric oxide production [33, 34], increased endothelin-1-mediated vasoconstriction [35, 36] elevated reactive oxidative stress [30, 37], enhanced inflammation [6, 38, 39], cerebromicrovascular rarefaction [37, 40-44], and cerebral microbleeds [45, 46]. Reduced expression of endothelial nitric oxide synthase (eNOS), the major enzyme producing nitric oxide in endothelial cells, results in spontaneous thrombotic cerebral infarction and cognitive impairments in aged mice [44]. Consistently, lack of redox enzymes, such as p66^Shc^ [30] and nicotinamide adenine dinucleotide phosphate oxidase 5 [47], in aged mice improves endothelium-dependent and nitric oxide-mediated relaxations in basilar arteries and prevents the development of memory deficits. In mice with endothelial-selective overexpression of endothelin-1, compared to the wild types, there is increased oxidative stress in the hippocampal region following ischemia/reperfusion challenge of the middle cerebral artery; this is associated with poorer cognitive function and more severe BBB breakdown [36]. Beta-site amyloid precursor protein cleaving enzyme (BACE) is a membrane-bound aspartyl protease [48], and its two isoforms, BACE1 and BACE2, are present in the brain endothelial cells. BACE1 cleaves amyloid precursor protein at the β-secretase site (the amyloidogenesis pathway), and BACE2 promotes the non-amyloidogenic processing of amyloid precursor protein like an α-secretase [49, 50]. The amyloidogenesis pathway results in the release of amyloid-β-peptide (Aβ) into the extracellular space, while the α-secretase-processing of amyloid precursor protein leads to a reduction of Aβ production. Indeed, increased plasma and cerebrospinal fluid levels of BACE1 are correlated to the progression of Alzheimer's disease in humans [51, 52]. In addition to processing amyloid precursor protein, endothelial BACE1, the expression of which is increased in cerebral vessels of hypertensive subjects, cleaves occludin in endothelial cells and disrupts tight junctions of the BBB, resulting in memory deficits [53]. Deleting BACE1 in mice improves microvascular, but not macrovascular, endothelial function by upregulating the expression of eNOS [54]. Moreover, BACE1 promotes the association between caveolin-1 and eNOS, resulting in reduced activation of eNOS. By contrast, BACE2, which is highly expressed in cerebrovascular endothelial cells, is downregulated in the endothelium of Alzheimer's patients [55]. BACE2 deficiency impairs the expression and function of eNOS in human brain microvascular endothelial cells, suggesting that BACE2 is a vascular protective protein [56]. However, nitric oxide, besides being a vasodilator, also modulates the expressions of tight junction proteins, such as occludin, zonula occludens-1 (ZO-1) and claudin-5, in mouse brain microvascular endothelial cells thereby affecting the BBB permeability [57-60]. In mouse brain microvascular endothelial cells exposed to Aβ, the activation of eNOS is reduced leading to reduced ZO-1 and claudin-5 expression [59]. To the contrary, increased eNOS-activation by vascular endothelial growth factor (VEGF) in mouse brain microvascular endothelial cells results in downregulation of occludin, ZO-1 and claudin-5, and increased tight junction permeability [60]. Examination of the effects of nitric oxide-releasing compounds on the permeability in rat brain and porcine brain endothelial cell monolayers suggests that high concentrations of nitic oxide cause BBB disruption [61, 62]. An increased release of nitric oxide from erythrocytes of old mice, when compared to those of young mice, results in reduced ZO-1 expression and increased barrier permeability in mouse brain endothelial cells [63]. Therefore, these findings suggest that the degree of eNOS activation needs to be tightly regulated in order for optimal functioning of the BBB.

Inflammation in endothelial cells also leads to the disruption of the integrity of the BBB. Intracellular levels of tumor necrosis factor (TNF) α and IL-1β in cerebrovascular endothelial cells are increased in 24-month-old mice when compared with those in the brains of 3-month-old mice [57]. Expressions of tight junction proteins, occludin-1 or ZO-1, are negatively correlated to the increased level of TNFα [57]. Complement precursor C3a cleaved from astrocytic C3 interacts with endothelial C3aR at the BBB, promotes upregulation of vascular cell adhesion molecule (VCAM)-1, and drives proinflammatory signaling and chemotaxis, resulting in immune cell infiltration in the brain of aged mice [58, 64].

Taken together, with aging, the metabolism and signaling of endothelial cells of the BBB are altered. These changes contribute to BBB leakage, leading to reduced glucose and blood supply to the brain and increased oxidative stress and inflammation, and are associated with impairment of cognitive function.

### 2.2 Pericytes

**Figure 1.**
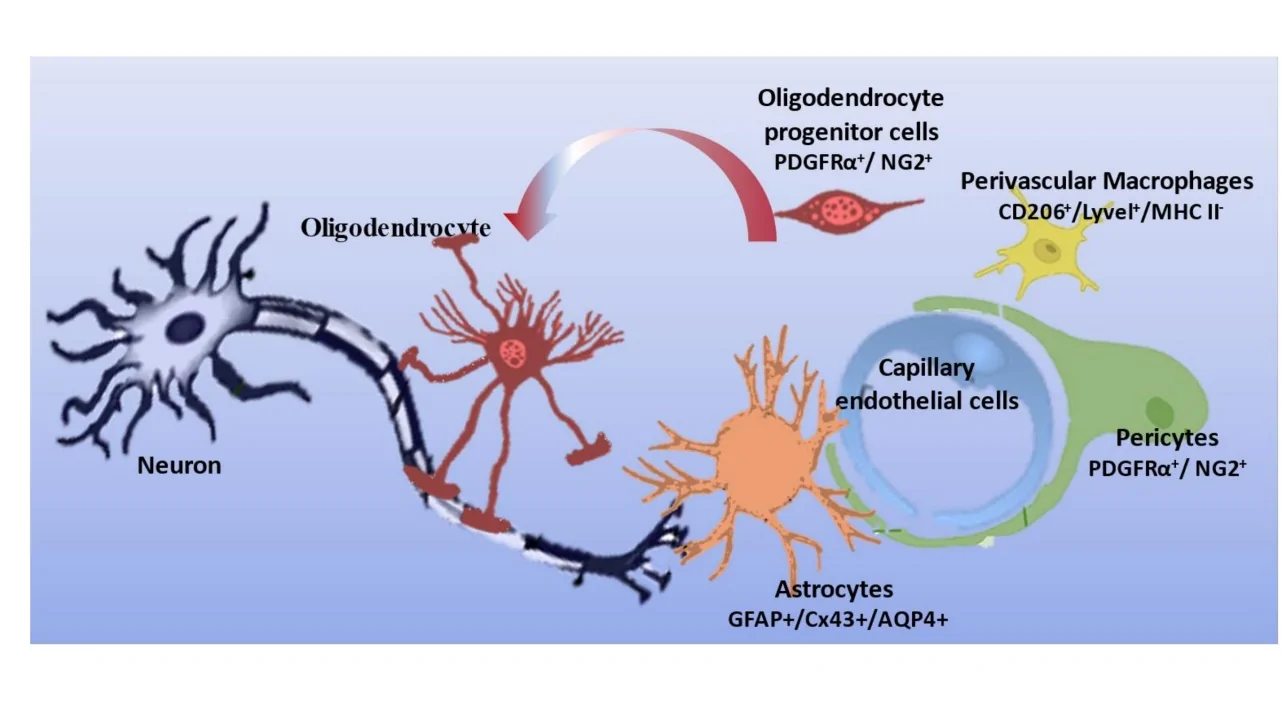
**The blood-brain barrier in the central nervous system**. The blood-brain barrier is composed of capillary endothelial cells, pericytes, and end feet of astrocytes. In addition, perivascular macrophages and oligodendrocyte progenitor cells are reported involved in the protection of the blood-brain barrier.

Pericytes are perivascular cells surrounding the vascular basement membrane with extended processes. They are characterized by ovoid cell bodies and long, thin processes with cell surface markers of chondroitin sulfate proteoglycan [CSPG4, also known as neuron-glial antigen 2 (NG2)] and PDGFRβ [65-68] (Fig. 1). Pericytes are in contact with the neighboring pericytes and endothelial cells, but not with the surrounding neurons or glia. They are an important component of the BBB, as demonstrated by the findings that pericyte loss, which occurs during aging, is associated with disruption of BBB integrity.

The role of pericytes in regulating cerebral blood flow is suggested by the presence of contractile proteins such as alpha-smooth muscle actin and myosin. Indeed, it is reported that noradrenaline (released by locus coeruleus) [69], reactive oxygen species [68] and optogenetic stimulation of gamma-aminobutyric acid interneurons [68] in rodents induce pericyte contraction, which in turn constrict cerebellar capillaries. Focal ablation of pericytes results in capillary dilation in mice, thus indicating that pericytes regulate a basal vasomotor tone on the cerebral vasculature [66, 68]. Pericyte contraction, at least following optogenetic stimulation, involves an influx of calcium through L-type calcium channels; and the increased intracellular calcium is taken up by mitochondria leading to reactive oxygen species generation and activation of Rho-kinase [68].

Pericytes relax to the neurotransmitter glutamate, which activates N-methyl-D-aspartate (NMDA) receptor in the neuron and subsequently nitric oxide synthase to release nitric oxide [69, 70], leading to capillary dilatation [65]. Notably, glutamate-induced capillary dilatation in rodents is attributed to nitric oxide-induced suppression of 20-hydroxyeicosatetraenoic acid formation, but not through activation of guanylyl cyclase to produce cyclic 3'-5' guanosine monophosphate (the main signaling molecule downstream of nitric oxide [12]) in pericytes [65]. Both glutamate and NMDA stimulate a hyperpolarizing current, likely due to potassium efflux induced by prostaglandin E_2_, in pericytes; and cerebellar capillary dilatation, being more prominent in areas where pericytes are present, is inhibited by blockade of EP_4_ receptors (receptors for prostaglandin E_2_) [65]. The findings thus confirm that pericytes actively relax in response to glutamate to cause capillary dilatation.

Reduced pericyte counts and coverage [37, 66, 71, 72], as well as low capillary density [41-43, 66], have been reported in aged rodent brains. By using an optical ablation approach, which damages pericytes but leaves microvascular endothelial cells undestroyed, local loss of pericytes induces capillary dilation. In aged mice, as well as under hypoxic conditions, this focal dilation results in severe flow steal [66, 73] and exacerbates flow heterogeneity in capillary networks compared with adult young mice. Some capillaries stall in flow and regress, leading to a loss of capillary connectivity. Furthermore, in aged mice, the remodeling of neighboring pericytes is impaired that pericyte coverage and capillary tone cannot be restored [66].

The crosstalk of pericytes with endothelial cells is important for the development and maintenance of the function of the BBB. Communications between pericytes and endothelial cells involve both physical contact through the gap junctions and via paracrine signaling. Through gap junction coupling, depolarizing and hyperpolarizing currents in pericytes propagate to endothelial cells to regulate the local vascular tone [70]. The paracrine signaling between pericytes and endothelial cells appears more complicated. On the one hand, angiogenic endothelial cells release platelet-derived growth factor (PDGF)-B, which binds to PDGFRβ on pericytes to stimulate pericyte proliferation and attachment to endothelial cells [41-43, 66]; the latter, in turn, supports the cerebrovascular stability [69, 70]. On the other hand, pericytes contribute to the regulation of endothelial transcytosis, as demonstrated by the findings that pericyte deficiency in rodents results in increased endothelial transcytosis and disrupted endothelial tight junctions, and hence increased BBB permeability [74-76]. Focal absence of pericytes in mouse brain is associated with BBB leakage, where the endothelial cell expressions of major facilitator superfamily domain containing 2A (a lipid transporter) and angiopoietin 2 (which contributes to the development of endothelial tight junctions) are reduced [76]. Moreover, in endothelial cells devoid of pericyte contact, the expression of intercellular adhesion molecules is upregulated; this is coupled with increased extravasation of leukocytes at the site, thus suggesting that pericytes also contribute to limiting neuroinflammation [74, 76, 77]. Exposure of porcine and human brain pericytes to inflammatory stimuli leads to inflammasome activation, and this further stimulates the release of inflammatory mediators and the loss of tight junctions in cerebral endothelial cells [78].

CD146, also known as the melanoma cell adhesion molecule, is reported as a cell surface marker of endothelial cells [79] and pericytes [80]. In particular, CD146 is present in the endothelial cells of immature microvessels without pericyte coverage, and in pericytes of the microvessels, but not in pericyte-covered endothelial cells in mouse brains [81], thus suggesting that CD146 plays an essential role in BBB formation during embryogenesis. Endothelial cell-specific knockout of CD146 downregulates claudin-5, but not ZO-1, protein expression, whereas pericyte-specific knockout of CD146 reduces pericyte recruitment to endothelial cells since the activation of PDGFRβ requires the direct interaction with CD146 [81]. Furthermore, endothelial expression of CD146 is reduced by pericytes-derived transforming growth factor-beta1 (TGF-β1), leading to reduced adhesion of leukocytes to endothelial cells [81].

Therefore, the loss of pericyte with aging leads to disrupted BBB structure and increased endothelial permeability. It is also an underlying cause of cerebrovascular inflammation and impaired brain capillary flow, both of which are characteristic features of aging-associated dementia.

### 2.3 Astrocytes

In the central nervous system, astrocytes are the main type of glial cells, which closely connect with neurons and extend elongated processes (end-feet) covering cerebral capillaries [82, 83] (Fig. 1). They are identified by their expressions of glial fibrillary acidic protein, connexin 43, and aquaporin-4 (GFAP^+^/Cx43^+^/AQP4^+^) and are responsible for secreting trophic factors and extracellular matrix proteins that support the BBB structure [82-84]. Connexin 43 (Cx43), a major membrane protein of astrocytes, forms gap junctions and hemichannels for cell-cell communication and exchange with extracellular space [84]. It is reported that in rat brain astrocytes challenged with oxygen-glucose deprivation/recovery, Cx43 is translocated from the plasma membrane to the cytoplasm; this results in disruption of astrocyte cell-cell adhesion and inhibition of the growth of adjacent neurons, implying that the membrane Cx43 is essential in maintaining the structure and function of astrocytes [85]. Aquaporin-4 (AQP4) is a membrane protein localized in the end-feet of astrocytes for regulating ion and water homeostasis in the brain. Its inhibition results in a greater accumulation of the injected Aβ around the blood vessels of the mouse brain [86]. It has been reported that with aging, there is a pathological accumulation of phosphorylated tau protein (a major mediator of the Aβ-neurotoxicity) in astrocytes. In a study of the Austrian elderly subjects, the protein presence of phosphorylated tau is increased, which is associated with the upregulation of Cx43 and AQP4 in astrocytes [87]. Together with the findings that Cx43 is involved in neuronal recovery [85] and AQP4 in Aβ clearance [86], these data suggest that increased Cx43 and AQP4 protein expressions are likely neuroprotective responses of astrocytes preceding pathological changes in the brain [87].

Astrocyte mitochondria are responsible for maintaining homeostasis by participating in glutamate and fatty acid metabolism, as well as by regulating calcium signals and reactive oxygen species generation [88]. By transferring their healthy mitochondria to the neurons, astrocytes protect neurons against oxidative stress-induced damage [89, 90]. However, with aging, astrocytes undergo senescence [84, 91], with increased production of reactive oxygen species [92], vulnerability to oxidative stress [91], and enhanced generation of proinflammatory mediators [84].

Inside the astrocyte, the mitochondria form physical contact with endoplasmic reticulum (ER) for metabolic function, oxidative metabolism, and calcium signaling [93]. Mitochondria and ER are clustered in the end-feet of astrocytes, and in mice subjected to cortical stab wound injury, the amount of mitochondria and ER in the end-feet increase markedly, suggesting that perivascular astrocytes are metabolically active and likely responsible for vascular remodeling [94, 95]. Indeed, by specifically deleing mitofusin 2 in astrocytes, which disrupts the mitochondria-ER contact, especially in the end-feet, endothelium-mediated angiogenesis, and hence neovascularization in the injured area, following cortical injury are impaired [94].

Astrocytes are also capable of producing endothelin-1, and the astrocyte-derived endothelin-1 is increased when mice are subjected to ischemic stroke caused by transient middle cerebral artery occlusion, resulting in progressive neurodegeneration [96]. Elevated levels of endothelin-1, in turn, stimulate the production and secretion of Aβ, which further simulates astrocytes to release soluble factors, including the inflammatory mediators (TNF-α, IL-6, IL-1β, monocyte chemoattractant protein-1 and C-X-C motif chemokine 10), VEGF, matrix metalloproteinases (MMPs), nitric oxide, and oxygen-derived free radicals. These soluble factors participate in downregulating junction proteins ZO-1 and claudin-5, and impairing angiogenesis by reducing the phosphorylation of eNOS (at serine 1177) and VEGF receptor 2 (VEGFR2) in mouse brain microvascular endothelial cells [59, 60]. Therefore, as astrocytes progress to senescent phenotype with aging, their neuroprotective ability to clear Aβ and promote vascular repair is reduced; more importantly, they contribute to neurodegeneration by increasing oxidative stress, Aβ production, inflammatory responses, and breakdown of endothelial tight junctions.

### 2.4 Perivascular macrophages

The perivascular macrophages (PVMs) are characterized by their expressions of CD206 and lymphatic vessel endothelial hyaluronan receptor-1 and lack of major histocompatibility complex II (CD206^+^/Lyvel^+^/MHC II^-^) [9, 10]. Examination of the movement of fluorescently tagged molecules with different size in the rat area postrema, where the BBB is less restrictive due to the absence of endothelial tight junction proteins claudin-5 and occludin, indicates that PVMs contribute to the maintenance of vascular barrier by taking up molecules greater than 10 kDa to limit their passage to the brain [97]. Moreover, PVMs play an important role in regulating immune responses during pathogen invasion in the central nervous system, based on the findings that their depletion results in reduced recruitment of neutrophils and increased bacterial counts in the cerebrospinal fluid in rats injected with the bacteria *S. pneumoniae* intracisternally [98]. Despite these neuroprotective effects, PVMs are implicated in the impaired cerebral blood flow response and cognitive dysfunction associated with hypertension. The pathological effects of PVMs are related to their expression of angiotensin type 1 receptors and NADPH oxidase 2, since depletion of PVMs prevents the reduced cerebral blood flow to endothelium-dependent vasodilator and memory deficits in spontaneous hypertensive mice and the neuroprotective effects of PVM-depletion are mimicked by neocortical application of angiotensin type 1 receptor antagonist or scavenger of reactive oxygen species [99]. The determination of the functions of PVMs is further complicated by the changes in transcriptional signatures of PVMs and the emergence of monocyte-derived macrophages in aging and neurodegenerative diseases, according to the studies analyzing the mouse brain PVMs using single-cell RNA sequencing combined with cytometry and fate-mating approaches [100]. Using a linage-tracing approach, the presence of CD206^+^/Lyve1^+^ PVMs is increased in a photothrombotic-induced stroke model, probably due to local proliferation rather than replacement by bone marrow-derived monocytes [100]. Therefore, it indicates that PVMs in brains are more heterogeneous than previously reported [10], which calls for further investigation to identify their functions under physiological and pathological conditions.

### 2.5 Oligodendrocyte progenitor cells

Oligodendrocyte progenitor cells (OPCs), also known as NG2 glia, are a subtype of glia in the central nervous system with surface markers of PDGFRα^+^ and CSPG4^+^ [101-103]. PDGFRα is critical for OPC maturation: in isolated rat and mouse OPCs, the activation of PDGFRα by low concentrations of its ligand PDGF is coupled to phosphoinositide 3-kinases-extracellular signal-regulated kinases signaling for cell migration [104, 105], while with high ligand concentrations, phospholipase C gamma signaling is activated for cell proliferation [104, 105]. In transgenic mice with increased release of PDGF-A by astrocytes, the number of OPCs counted in the adult central nervous system, especially in white matter tracts, increased [106].

OPCs are located close to endothelial cells, which release VEGF to promote the survival and proliferation of OPCs. In turn, OPCs mediate the function of endothelial cells. When rat brain endothelial cells are co-cultured with OPCs, endothelial cell permeability is reduced; this decrease is prevented by blockade of PDGFRα in the OPCs, thus suggesting that communication between brain endothelial cells and OPCs play a role in the maintenance of BBB integrity [107]. The obligatory role of OPCs is to replenish damaged oligodendrocytes for the regeneration of myelin sheath and to maintain microglia homeostasis under pathological conditions [108-110]. With aging, OPCs have reduced capacity for cholesterol biosynthesis, leading to impaired differentiation into oligodendrocytes and blunted remyelination [103, 111], myelin destruction [111, 112], and oligodendrocyte death [113]. Immuno-fluorescence staining of postmortem brain tissue section of patients with Alzheimer’s disease demonstrates that the major cell type associated with the Aβ plaques are OPCs with a senescence phenotype [114]. Moreover, the neurotoxin Aβ causes mouse OPCs to undergo senescence; the Aβ-associated senescent OPCs in a mouse model of Alzherimer’s disease exhibit a proinflammaory phenotype, and oral administration of these mice with the senolytic cocktail of dasatinib and quercetin results in removal of senescent OPCs, downregulation of neuroinflammation and amelioration of the cognitive deficits [114].

Both increased counts of OPCs and decreased counts of mature oligodendrocytes are observed in microvascular brain injury patients, suggesting that myelination disturbances in these patients are related to the disrupted differentiation of OPCs to myelinating oligodendrocytes [32]. In addition to endothelial cells, astrocytes, microglia, and pericytes also contribute to the regulation of the differentiation of OPCs to oligodendrocytes [115, 116]. In the differentiation stage, the expression of a disintegrin and metalloproteinase with thrombospondin motifs (ADAMTS)-4, the most expressed proteoglycanase among the ADAMTS family in the central nervous system [117], in the OPCs is increased [118]. One of the substrates of ADAMTS-4 is NG2, a surface marker of OPCs [102, 103]. In mice with genetic ablation of ADAMTS-4, NG2 proteolysis is prevented, leading to elevated PDGFRα signaling and impaired oligodendrocytes differentiation and neuronal myelination for brain development and for myelin repair after injury [118]. The expression of ADAMTS-4 is increased in cultured human astrocytes and mouse microglia by the inflammatory mediator TNFα, as well as in the brains of human and rodents with stroke [119, 120]. With increased ADAMTS-4, the expression of NG2 and activation of PDGFRα are reduced, and oligodendrocyte differentiation is accelerated [118]. However, neonatal rat OPCs exposed to the lysed microglia from aged rats, or to lysed microglia from young rats activated by TGF-β (the circulation of which is increased in aging), are differentiated into astrocytes rather than oligodendrocytes [115]. Likewise, the depletion of A-kinase anchor protein 12 (AKAP12) on pericytes in mice results in impaired OPC-to-oligodendrocytes differentiation and reduced myelin density in the central nervous system [116].

## 3. Pathological changes and the mechanism leading to aging-associated cognitive dysfunction

**Figure 2.**
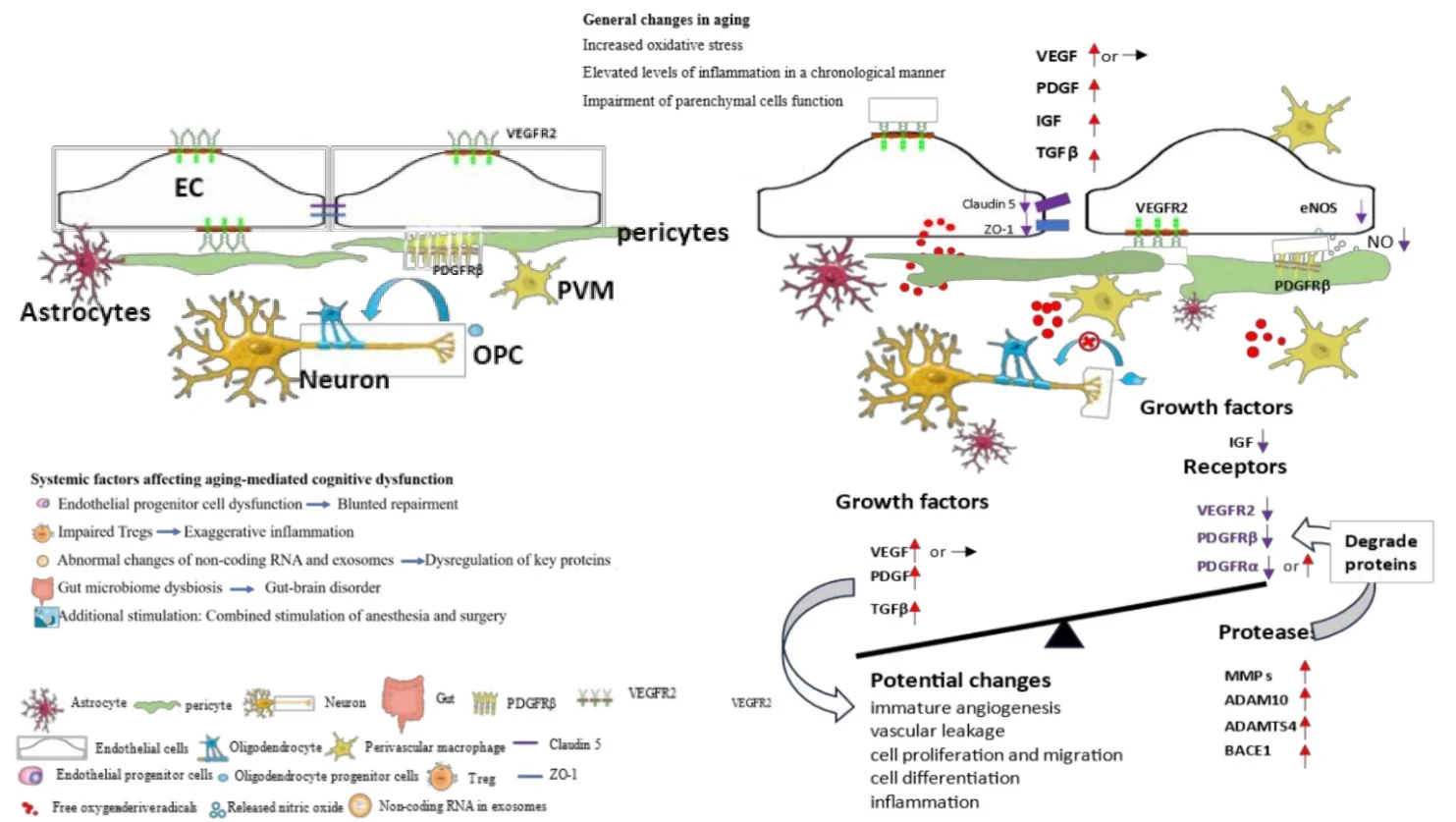
**The organization of the blood-brain barrier under normal (*Left*) and pathological conditions (*Right*)**. Aging is an unavoidable physiological event, causing structural and functional changes in the blood-brain barrier, resembling many pathological changes under disease conditions. In the process of aging, oxidative stress and serum inflammatory levels are increased in a chronological manner, accompanied by parenchymal cell dysfunction. Impaired endothelial progenitor cells blunt microvascular repairment, aged Tregs exaggerate proinflammation, and non-coding RNAs dysregulate key proteins in the blood-brain barrier. External impacts, including anesthetic exposure, surgery, and emotional breakdown further disrupt the defense of the blood-brain barrier. Indeed, eNOS expression and its product NO are reduced. Tight junction protein presence is downregulated. Pericyte counts and coverage are decreased. Thus, oxidative stress is increased in the central nervous system, leading to increased presence of perivascular macrophages and activated astrocytes. Of importance, growth factors PDGF, VEGF, and TGFβ are increased in the central nervous system. However, their corresponding receptors are reduced, which is attributed to increased expressions of proteases in aging, including MMPs, and ADAM10. ADAMTS4, and BACE1.

From the above discussion, it appears that during aging, the cells compositing the BBB have increased oxidative stress and inflammatory responses, which lead to the disruption of the integrity of the BBB and hence impaired cognitive function (Fig. 2). These changes are partly the result of age-associated dysfunction of the cells in the BBB, and partly due to the presence of harmful systemic soluble factors which circulate to and subsequently increases the permeability of the BBB. The latter appears to underlie the condition known as postoperative cognitive disorder (POCD), with patients displaying impaired learning capacity, memory loss, confusion, anxiety, and personality changes after major surgery and is attributed to exposure to anesthetics and/or surgical procedures [121]. It is reported that increasing age in the patients (those aged 70 years or older, compared to those 60 to 69 years old) is associated with early (first week) and long-term (3 months) POCD [121]. Propofol, isoflurane, and sevoflurane are commonly used anesthetics in clinical practice, and several animal and clinical studies have suggested that their use is associated with the occurrence of POCD [122-125]. Propofol increases the BBB permeability, and this effect is enhanced by advanced glycation end products, which are accumulated in diabetes, as demonstrated by the increased bacterial traversal in an *in vitro* BBB model [122]. Exposure to isoflurane leads to the disruption of BBB through the upregulation of VEGF in the hippocampus of aged mice [126, 127]. The neurotoxicity associated with sevoflurane involves an enhanced inflammatory response associated with microglial activation and upregulation of a major proinflammatory transcription factor, nuclear factor-kappa B (NF-κB) [128, 129].

### 3.1 Protein expression changes in the cells of the BBB during aging

With aging, the expressions and/or activities of several proteins in astrocytes, endothelial cells and pericytes are altered. Among them, changes in the expressions/ activities of the cleavage enzymes, MMPs and caspases, as well as the inflammatory mediators, IL-33 and transactive response DNA-binding protein of 43 kDa (TDP-43), appear to be involved in the development of aging-associated cognitive dysfunction.

MMPs, belonging to a large family of proteases, play an essential role in degrading extracellular matrix proteins. In mice with ischemia stroke [34, 130], exposed to anesthesia and surgery [131], or fed with high-salt diet [60], MMPs are activated, leading to the breakdown of the BBB [132]. In particular, tight junction proteins of BBB, such as ZO-1, claudin-5, and occludin, are sensitive to degradation by MMP-2 and MMP-9, which are expressed and released by activated astrocytes and microglia [131, 133, 134]. In aged mice [135] and aged mice with POCD [131], inhibition of MMP-2/-9 increases tight junction protein expression, inactivates microglia and astrocytes, and restores cognition. MMP-2 is present in astrocytic end feet surrounding cerebral blood vessels while the expression of MMP-9 and MMP-12 in the mouse brain are upregulated with aging [136]. Unlike that of MMP-2, the promoter gene sequence of MMP-9 and MMP-12 contain the binding sites for the transcription factors NF-κB and activator protein-1; as such, MMP-9 and MMP-12 are upregulated during inflammatory responses and contribute to neuroinflammation by promoting the infiltration of T-lymphocytes and bone marrow-derived microglia in the brain [130, 136]. By proteolytic cleavage, MMP-2/-9 activates TGF-β, increases chemokines secreted by astrocytes, and enhances the production of chemokines [137]. The expression of MMP-9 in cultured mouse microglia exposed to the bacterial endotoxin, lipopolysaccharide, is reduced by ADAMTS-4, which is upregulated by TNFα stimulation, thus suggesting a role of ADAMTS-4 in regulating BBB permeability during neuroinflammation [119]. Besides the tissue inhibitors of metalloproteinase, caveolin-1 in brain microvascular endothelial cells also contributes to the regulation of MMP activity [138]. It has been reported that caveolin-1 negatively regulates nitric oxide production by physically interacting with nitric oxide synthase (all three isoforms) via the caveolin scaffold domain [139], which is also present in the gelatin-binding and hemopexin-like domains at the carboxyl-terminal of both MMP-2 and MMP-9 [140]. In line, knocking down the expression of caveolin-1 in rat brain microvascular endothelial cells results in increased MMP activity; since nitric oxide mediates the upregulation of caveolin-1 in rat brain microvessels following focal cerebral ischemia-reperfusion injury, inhibition of nitric oxide production reduces BBB permeability by indirectly inhibiting MMP activity [138].

Caspases are a family of protease enzymes playing essential roles in programmed cell death. A total of 14 caspases has been reported, among which caspase-6 is activated in patients with age-dependent cognitive impairment and Alzheimer's disease [141, 142]. Overexpression of caspase-6 leads to cognitive deficits and astrocyte activation in aged mice, while inhibition of caspase-6 by methylene blue restores cognition function after the onset of cognitive deficits [143]. The activation of caspase-6 in astrocytes is reduced by deletion of caspase-9 in retinal endothelial cells; as a result, astrocyte activation is inhibited and the level of inflammatory cytokines is downregulated, thereby preventing neuronal function decline in a mouse retinal vein occlusion model [144]. Caspase-9 plays a role in maintaining mitochondrial homeostasis [145], cytokines activation [144], myocyte differentiation and proliferation [146], and insulin-like growth factor (IGF)-2 receptor retrieval [147], which are independent of its role in apoptosis. Activation of caspase-9 and its downstream effector caspase-7 in endothelial cells is observed in mouse retinas subjected to retinal vein occlusion, resulting in endothelial barrier leakage and prolonged vascular occlusion with the consequence of neuronal injury [148].

In the aged population, the incidence of POCD is higher [121, 149]. The higher occurrence of POCD in aged animal models contributes to chronic systemic inflammation, disruption of the BBB, enhanced inflammation in the central nervous system, and impairment of neuronal function [131, 150, 151]. Downregulation of IL-33 and its corresponding receptor, suppression of tumorigenicity 2 (ST2), is observed in the hippocampus of mice subjected to anesthesia and surgery [152]. IL-33 is a member of the IL-1 family with a high expression in the central nervous system compared to other organs and functions to induce immune cells to produce type 2 cytokines (including IL-4, IL-5, IL-6, IL-10, and IL-13) [153]; as such, activation of ST2 by IL-33 results in protective effects against cognitive impairment caused by POCD [152] and stroke [154] in mice. Indeed, IL-33 treatment reduces inflammatory responses in the central nervous system, shifts microglia polarization towards an anti-inflammatory phenotype, and increases the excitatory (glutamatergic) synapse density in the hippocampus, resulting in cognitive improvement in mice subjected to anesthesia and surgery [152, 155]. In a small group of patients with amnestic mild cognitive impairment and Alzheimer's disease, the presence of IL-33 is associated with better cognitive function over a year, while the levels of Aβ, tau, and apolipoprotein E-ε4 (a genotype known to associated with cognitive decline) are comparable between patients expressing and those not expressing IL-33 [156]. By contrast, it is also reported that increased expression of IL-33 in mice, either by exogenous administration or induced by experimental cerebral malaria, results in microglial activation and IL-1β production, depicting a harmful effect of IL-33/ST2 axis in cognitive function [157-159]. IL-33 is upregulated in endothelial cells and triggers pathological neovascularization through activating NF-κB-mediated Jagged1 signaling leading to deubiquitination of Notch1 in mouse hypoxic retinas [159]. In mouse cerebral microvascular endothelial cell, IL-33 increases the protein levels of adhesion molecules, VCAM-1, P-selectin and E-selectin, subsequent to the activation of p38 mitogen-activated protein kinase and extracellular signal-regulated kinases [160]. Thus, the role of IL-33 in cognitive preservation /dysfunction warrants further investigation. TDP-43, a transcriptional repressor, is a structural hallmark of neurodegenerative diseases. Elevated neuronal TDP-43 induces activation of microglia, astrocytes, pericytes, and endothelial cells, resulting in T cell infiltration into the brain (without changes in the tight junction protein level) and impaired cognition in mice [161].

### 3.2 Circulating factors affecting the function of the BBB during aging

PDGF is the ligand to PDGFRα and PDGFRβ, characteristic markers of OPCs and pericytes, respectively [65-68, 101-103]. In aged mice, the serum level of PDGF increases; this is associated with the shedding of PDGFRβ in pericytes, leading to pericyte loss and impairment of hippocampal vasculature [67]. By contrast, in cultured human brain pericytes, PDGF-BB reduces cell apoptosis and stimulates cell proliferation [162] and intravenous treatment with PDGF-BB peptide promotes pericyte coverage and restores capillary tone and flow rate in a mouse epilepsy model [163]. Therefore, it appears that aged pericytes have altered response to PDGF.

VEGF is a family of growth factors specifically affecting vascular endothelial cells due to their expression of VEGFR2, including increasing vascular permeability, promoting cell proliferation, migration, angiogenesis, and inhibiting cell apoptosis [164]. Increased level of VEGF, which are released in the central nervous system under unfavourable conditions, for example, by OPCs exposed to hypoxia [165] and astrocytes exposed to high salt environment [60], lead to the destruction of tight junctions in the endothelial cells [60] and immature angiogenesis [166]. On the other hand, in postnatal mouse brain, VEGF is mainly expressed in neurons; deletion of brain VEGF results in ablation of blood vessels and impaired neuronal development in postnatal mice [167]. In a longitudinal monitoring study in mice, VEGF levels in the plasma and major peripheral organs are not significantly decreased with aging [168]. However, the activation of the VEGFR2, reflected by its phosphorylated levels at tyrosine 1175 residue, is reduced, due to the trapping of VEGF by the soluble VEGF receptor 1 (sFlt1), a decoy receptor [168]. These data indicate that VEGF signaling is compromised in aging with the resultant capillary rarefaction and hypoxia in tissues. Indeed, VEGF induction in the brain promotes endothelial cell proliferation with increased microvascular density and attenuates age-related neurogenic decline in mice [169]. These findings, therefore, suggest that VEGF, particularly those released by neurons, is essential for the development and maintenance of vascular and neuronal networks; by contrast, increased production of VEGF, likely from activated OPCs or astrocytes, would result in BBB dysfunction and hence cognitive deficits [166].

IGF-1 is critical for brain development and neurogenesis. Both astrocytes and endothelial cells of the BBB express IGF-1 receptors (IGFR). IGF-1 reaches its highest production in the pubertal stage and decreases in aging [170-172]. With the reduction of circulating IGF-1 level during aging, the structure and function of the neuro-glia-vascular unit are impaired [173]. Exposure of cultured rat brain microvascular endothelial cells to IGF-1 prevents the oxygen-glucose deprivation/recovery-induced increase in permeability [174]. In mouse thymic endothelial cells, IGF-1 promotes angiogenesis [175]. The impairment of cerebral blood flow in aged brains is related to blunted IGF-signaling since it can be mimicked by endothelial-selective deletion of IGFR in mice [176]. Blockade of IGFR in rodents reduces the presence of glutamate transporters in astrocytes [177], leading to impaired glutamate cycling and increased glutamate excitotoxicity in neurons [178].

TGF-β belongs to the transforming growth factor superfamily, including TGF-β1 to -β3, among which TGF-β1 level in the brain is increased in vascular dementia and Alzheimer's disease [179]. A possible source of TGF-β in the brain of mice subjected to cortical stab wound injury is the deposition of fibrinogen carrying the latent TGF-β from the systemic circulation under pathological conditions [180]. The released TGF-β is activated by the astrocytes in the brain, leading to Smad2 protein phosphorylation in astrocytes and neurite outgrowth arrestment [180]. Human brain pericytes also release TGF-β1 and the release is increased following exposure to advanced glycation end-products [181]. TGF-β1, in turn, stimulates fibronectin production in pericytes, leading to basement membrane hypertrophy and BBB disruption [181]. Moreover, TGF-β1 upregulates the expression of mitogen-activated protein kinase phosphatase-1 in cerebrovascular smooth muscle cells, thereby impairing endothelin-mediated vasoconstriction by inhibiting the signaling of p38 mitogen-activated protein kinase [182]. TGF-β also prevents the differentiation of rat OPCs to oligodendrocytes, resulting in myelination disturbance in the central nervous system [115]. On the other hand, deletion of TGF-β1 or its downstream signaling molecules in mouse endothelial cells leads to reduced differentiation of endothelial to mesenchymal cells thereby restoring interaction between endothelial cells and pericytes and hence minimizing BBB breakdown during cerebrovascular pathologies [183]. By contrast, deletion of TGF-β signaling in mice results in blood vessel malformation and embryonic lethality due to an improper attachment between endothelial and mesothelial cells [183]. These findings, therefore, suggest that proper TGF-β signaling is important in the maintenance of BBB. TGF-β signaling is regulated by high-temperature requirement A serine peptidase (Htra) 1, the gene expression of which is downregulated in an age-dependent manner [46]. There is no consensus on how Htra1 regulates TGF-β signaling: Htra1 suppresses TGF-β signaling by cleaving TGF-β precursor/receptors [184, 185] or activates it by cleaving the latent binding protein of TGF-β [186]. Thus, mechanisms underlying the interaction between Htra1 expression and TGF-β signaling in cerebral endothelial cells need to be further investigated.

Gut bacteria release many bioactive compounds into circulation, including short-chain fatty acids and bile acid metabolites, and these compounds have been shown to affect the BBB function and neuroinflammation [187, 188]. As such, gut microbiome dysbiosis has significant impacts on cognitive function [187, 188], as well as on intestinal hemostasis [189], medicine bioavailability [190, 191], and immune function [192]. In particular, the plasma level of the gut microbiome-derived metabolite trimethylamine N-oxide is increased with aging and is negatively correlated with cognitive function in the aged healthy population [193]. Further study in mice indicates that trimethylamine N-oxide induces inflammation in the brain by activating astrocytes [193].

Albumin is the most abundant protein in plasma. It infiltrates into the extravascular space in the central nervous system following BBB disruption which occurs in aging and under pathological conditions, such as traumatic brain injury, ischemic stroke and Alzheimer’s disease [7, 8]. In rats treated with BBB-disrupting agent, albumin is taken up by astrocytes through the TGF-β receptors, resulting in increased accumulation of extracellular potassium which further causes NMDA receptor-mediated neuronal hyperexcitability [194]. While albumin does not directly affect the degree of synapse in isolated rat neurons, it activates TGF-β receptors in astrocytes to stimulate the generation of excitatory synapse in rat neurons [195]. Besides inducing neuronal excitability, albumin increases the expression of myosin light chain kinase and MMP-9 in isolated rat astrocytes; these effects are mediated through activation of p38 mitogen-activated protein kinase and contribute to increased BBB permeability [196, 197]. It also stimulates isolated mouse microglia to release inflammatory mediators, which, in turn, activate astrocytes to produce very long-chain saturated fatty acids to cause neuronal apoptosis [198]. By increasing BBB permeability and inducing neuroinflammation and neuronal apoptosis, the presence of albumin in the central nervous system would lead to cognitive dysfunction, as observed in mice receiving intracerebroventricular injections of albumin [198].

Red blood cells adopt a different structure with an impaired integrity with aging, based on the findings that red blood cells isolated from the elderly showing greater cell damage and greater release of hemoglobin (a major protein in the red blood cells) under physiological shear stress than those isolated from the young individuals [63]. Moreover, red blood cells from old, but not young, human and mice increase the permeability of an *in vitro* BBB model, which is made up of cocultured mouse brain microvascular endothelial and astrocytic cells, and this effect is caused by increased eNOS-mediated nitric oxide release from old red blood cells causing the downregulation of endothelial ZO-1 [63]. Systemic hemoglobin is also shown to disrupt BBB in guinea pig, and this effect is associated with increased oxidative stress and reduced ZO-1 expression in endothelial cells, as well as increased iron deposition in the brain [199]. With a leaky BBB, hemoglobin becomes present in the central nervous system where it causes further disruption of BBB, as shown in rats that intracerebroventricular injection of hemoglobin increases the expression and activity of MMP-9 in the blood vessels through activation of Rho-kinase and production of peroxynitrite, leading to reduced expressions of tight junction proteins and increased BBB permeability [200, 201]. The presence of hemoglobin and iron in rat brain also led to, respectively, NF-κB-induction in microglia and lipid peroxidation coupled with neuronal death [199, 200, 202]; these consequences likely result in cognitive impairment.

Regulatory T cells (Tregs) contribute to immune homeostasis as they suppress excessive immune responses [203]. Tregs increase IL-6 and fibroblast growth factor (FGF)-β and promote myelin production in rat OPCs exposed to oxygen-glucose deprivation/ recovery *in vitro* [204]. By contrast, in aged mice subjected to surgery, Tregs aggravate the disruption of BBB, resulting in increased production of TNFα in the hippocampus; such effects are partly attributed to the aging of the Tregs with impaired function [150].

Endothelial progenitor cells (EPCs) are an important player in the regeneration of the endothelial lining of blood vessels [205]. They are defined as circulating cells expressing surface markers similar to those expressed in endothelial cells. Compared with young people, circulating EPCs decreased in aged subjects [206], accompanied by reduced sirtuin 1 protein and increased acetylation of p53 protein in EPCs [207]. Aged EPCs exhibit impaired migration and reduced secretion of pro-angiogenic factors [207].

## 4. Potential therapeutic strategy for aging-mediated cognitive impairment

There is no doubt that cognitive impairment develops with aging. Therefore, approaches to improve general health during aging, including healthy lifestyle, physical exercise, and diets rich in resveratrol, such as peanuts, pistachios, grapes, and wine, are likely to be beneficial in retarding the progression of cognitive impairment [1, 2, 16]. Other more specific approaches have been proposed, and some of them are based on the understanding of the pathological factors leading to cognitive dysfunction relating to the cell components of the BBB, as indicated below.

### 4.1 Cell-based therapies

Given the importance of brain endothelial cells in the maintenance of BBB integrity, cell therapy with EPCs has been proposed as a potential barrier repair strategy [208-210]. As an analogy of the BBB, the effects on the blood-spinal cord barrier after transplanting bone marrow EPCs (CD15^+^/CD90^+^CD105^+^/CD106^+^/CD117^+^/CD309^+^) have been examined in mice with symptomatic amyotrophic lateral sclerosis. Following intravenous administration, bone marrow EPCs are engrafted into capillaries replacing the damaged endothelial cells, resulting in upregulated junction protein expressions and reduced capillary leakage. Their differentiation into endothelial cells is caused by the increased secretion of the angiogenic factors, VEGFA and angiogenin-1 (Angpt1). It further leads to the re-establishment of the pericyte coverage and perivascular astrocyte end-feet in the damaged blood-spinal cord barrier, as well as the promotion of spinal cord motor neuron survival [208-210]. Adiponectin is derived from adipocytes, exerting protective effects on vascular endothelium [211] and neurons [212, 213], and its low plasma level is associated with cognitive impairment in postmenopausal women and prediabetic patients [212, 213]. Consistently, treatment of adiponectin-transfected EPCs increases microvessel density, reduces proinflammatory cytokine levels, and improves cognitive function in aged rats [214].

Mesenchymal stem cells are a subgroup of non-hematopoietic stem cells in bone marrow; they support the differentiation of hematopoietic stem cells in the bone marrow and facilitate the growth and movement of stem cells in other tissues [215]. Unlike the intravenous transplantation of EPCs, mesenchymal stem cells barely trespass the BBB. Nevertheless, intracranial injection of mesenchymal stem cells restores the central nervous system function, protects against BBB disruption, and downregulates inflammation in the central nervous system in ischemia-induced stroke in rodents [216, 217]. Mesenchymal stem cells with the overexpression of fibroblast growth factor 21 are more effective than those without the overexpression in protecting against ischemia stroke-induced BBB disruption in rats, shown as greater upregulation of tight junction proteins, occludin, claudin-5, and ZO-1, as well as greater downregulation of AQP4 and MMP-9 [217]. Furthermore, mesenchymal stem cells exert their protection on the BBB through the release of TNF-α stimulated gene/protein 6, which suppresses the NF-κB signaling pathway and reduces the production of oxygen-derived free radicals in the rat brain subjected to intracerebral hemorrhage [218].

### 4.2 Cell-free therapies

Micro-RNA (miRNA), belonging to the non-coding RNA family, regulates target genes by binding to 3’-untranslated regions of mRNA sequences to induce gene silencing. Therefore, miRNA therapy offers protection against BBB dysfunction by preventing the expression of pathological signals involved in the damage. Pericyte-derived miRNA-210-5p regulates endothelial barrier function by inhibiting Janus kinase 1/ signal transducer and activator of transcription 3 signaling, promotes the recovery of motor function, and protects the blood-spinal cord barrier in mice after spinal cord injury [219]. MiRNA-181a, by inhibiting the FOXO1 expression, reduces pericyte loss and BBB breakdown with improved cognitive function in aged mice with Alzheimer's disease pathology [220]. MiRNA-671-5p and miRNA-23a-5p preserve BBB integrity in mice following ischemic stroke by downregulating brain endothelial NF-κB/MMP-9 [130] and astrocyte TNF/MMP-3 pathways [221]. respectively. MiRNA-195, which is present in extracellular vesicles secreted from astrocytes, protects against age-related BBB leakage in mice through the autophagic-lysosomal pathway by downregulating the expression of thrombospondin-1 in cerebral endothelial cells [222].

Extracellular vesicles are lipid-bound vesicles containing cellular signals, including proteins and mRNA, and are released by many cells for cell-cell communication. In an *in vitro* study, the nanosized vesicles derived from EPCs are taken up by mouse brain endothelial cells, protecting them from the damage induced by the serum from amyotrophic lateral sclerosis mice [223]. By using mass spectrometry proteomic analyses, microvesicles derived from mesenchymal stem cells are highly enriched for proteins involved with exosome biogenesis, vesicle trafficking, Rab-related GTPases, anti-apoptosis/programmed cell death, as well as neuron differentiation/neurogenesis. Therefore, it is reasonable to predict that mesenchymal stem cell-derived microvesicles exert protective effects in the central nervous system as the cells [216].

Probiotic treatment of *Lactobacillus* has also been proposed as a potential therapy to preserve BBB integrity with aging, based on the report of a negative correlation between the plasma level of gut microbiome-derived metabolite and cognition function in the aged population [193]. In aged mice subjected to anesthesia and surgery, administering *Lactobacillus* mix or the corresponding metabolite sodium butyrate increases junction protein and reduces BBB permeability [224].

### 4.3 Commercially available medicinal compounds

In addition to the above innovative approaches awaiting clearance from safety concerns and scientific evidence for effectiveness in human studies, some currently available medications have been shown to have protective effects on BBB and cognitive function.

Metformin, a first-line pharmacological treatment of diabetes, has the pleiotropic effect of protecting against cognitive dysfunction in individuals with and without diabetes [225]. Both 5‘-monophosphate-activated protein kinase (AMPK)-dependent [226, 227] and -independent [228] mechanisms are involved in metformin-exerted beneficial effects in the neuro-glia-vascular unit. Upregulation of AMPK restores junction protein occludin and claudin-5 expression by reducing oxidative stress in cultured human brain microvascular endothelial cells exposed to lipopolysaccharide [229]. Metformin enhances remyelination by restoring the differentiating potential of OPCs from aged rats [230] and inhibits neuronal apoptosis by inhibiting the mitochondrial fission protein Drp1 in neurons of diabetic mice [228].

Glucagon-like-peptide-1 (GLP-1), a meal-stimulated hormone by ileum neuroendocrine L-cells, stimulates insulin release and inhibits glucagon release, and is another class of drugs for diabetic treatment. GLP-1 has direct protective effects on rat endothelial cells under hyperglycemic/diabetic conditions [231-233]. In aged mice, the transcriptomic changes in brain endothelial cells and the BBB leakage are reversed by exenatide, a short-acting GLP-1 agonist [27].

Fluoxetine is used clinically as an antidepressant by selectively inhibiting the uptake of serotonin [also known as 5-hydroxytryptamine (5-HT)] into neurons. Fluoxetine has been shown to improve cognition and memory in patients with psychiatric symptoms and Alzheimer's disease [234] through inactivating glycogen synthase kinase 3β in OPCs/oligodendrocytes, preventing cell senescence and promoting the formation of myelin [235, 236]. Nevertheless, the use of fluoxetine in healthy aging people would not be advocated due to its antidepressant effects.

## 5. Future perspectives

Over the years, a vast body of research has been conducted to provide an insight in the etiology of the dysfunction of BBB during aging, with the aim to identify effective measures to prevent or retard the progression of cognitive impairment. Current knowledge on the pathological processes is mainly based on findings from studies in rodents, and cautions are warranted in extrapolating these findings to human conditions. Since the central nervous system is located inside the skull, it is barely accessible through any non-invasive medical approaches. As such, there are only limited studies in humans; among those, the number of participants is small, and only serum/plasma levels of the markers for neuronal damage and proinflammatory mediators are measured [51, 52, 156, 193, 206]. Therefore, the findings from the limited human studies may not be representative of the general population and permit only the determination of the relationship between changes in the systemic circulation and cognitive dysfunction without any mechanistic information. While the dynamic contrast-enhanced magnetic resonance imaging has been utilized to detect BBB leakage by measuring fluorescent tracing changes in different brain areas in living humans [8], tissue samples can only be obtained from post-mortem human brain from brain banks with consent [32, 55, 87]. Due to ethical issues, experiments on live human tissues, especially those for healthy controls, are not feasible. Future research would require the successful differentiation of human glial cells from stem cells for studying the signaling pathways inovlved in the disease process, and the development of brain organoids derived from human cells in order to study the complex crosstalks among the different cell types for the maintenance of the BBB integrity.

As mentioned above, not only changes in the central nervous system (section 3.1) but also changes in the components (for example, the levels of growth factors and gut microbiome-derived metabolites) or the characteristic of the cells (for example, red blood cells, Tregs and EPCs) in the systemic circulation (section 3.2) during aging can lead to BBB disruption. It is also recognized that cognitive dysfunction is exaggerated under certain systemic diseases and so mechanistic studies of cognitive imparirement have been conducted in animals with hypertension [99] or in glial cells exposed to bacteria [98], bacterial endotoxin [119] and advanced glycation end products [122, 181]. Thus, interdisciplinary research covering cardiovascular, immunology, hematology and endocrinology are required for advancing the knowledge on preserving the BBB integrity and cognitive function with aging.

## 6. Conclusion

The BBB is the anatomic and biological defense for the central nervous system. Aging-induced endothelial dysfunction and pertinent molecular changes affect other structural cells in the BBB and parenchymal cells in the central nervous system (Figure 2). Given the complexity of the BBB involving the interaction among the different cell types, the full picture of how the BBB integrity is maintained remains elusive. Current strategies against aging-mediated cognitive impairment are mainly for the general anti-aging approaches. The innovative concepts of replacing damaged cells in BBB using progenitor cells and/or stem cells and protecting BBB cells from developing into senescent/apoptotic phenotypes by non-coding miRNAs require further investigation, and the challenges of safe and non-invasive delivery of these cells/miRNAs to the BBB would need to be addressed. Some current medications appear to have pleiotropic effects to preserve BBB function, and their ‘therapeutic’ effects may become a hindrance since these may pose adverse effects in the healthy aging population. Currently, the BBB cell-derived extracellular vesicles and healthy gut microbiota seem promising. Further studies are needed for better understanding the interplay among different BBB cell types and the pathological cause of BBB disruption to shed some light on preventing aging-mediated cognitive dysfunction.
